# Anti-lipid droplets accumulation effect of *Annona montana* (mountain soursop) leaves extract on differentiation of preadipocytes

**DOI:** 10.32604/biocell.2022.014009

**Published:** 2021-11-18

**Authors:** Ivy LEUNG, Maria-Luisa VEISAGA, Margarita ESPINAL, Wei ZHANG, Robert BARNUM, Manuel Alejandro BARBIERI

**Affiliations:** 1 Department of Biological Sciences, Florida International University, Miami, 33199, USA; 2 Biomolecular Sciences Institute, Florida International University, Miami, 33199, USA; 3 Fairchild Tropical Botanic Garden, Coral Gables, 33156, USA; 4 International Center of Tropical Botany, Florida International University, Miami, 33199, USA

**Keywords:** *Annona montana* extracts, Phytochemical analysis, Adipogenesis, Lipid droplets, Adipogenic-specific markers

## Abstract

The *Annona* genus is a member of *Annonaceae,* one of the largest families of plants across tropical and subtropical regions. This family has been used in several ethnomedicinal practices to treat a multitude of human diseases. However, the molecular mechanism underlying its effect on the lipid droplet formation and on the expression of adipogenic markers of this plant remain to be investigated. In this study, we examined whether the extracts from the aerial part of *Annona montana* affect *in vitro* differentiation of preadipocytes. For our investigations, both mouse embryo fibroblast 3T3-L1 and normal human primary subcutaneous preadipocytes were incubated with *Annona montana* extracts (-and its subfractions-) and then analyzed on preadipocyte differentiation, lipid content, lipid droplet size and number, the expression of adipogenic-specific transcriptional factors, as well as cell survival. From our examinations, we found the *Annona montana* ethyl acetate extract to exhibit a potent inhibitory effect on adipogenesis, without affecting cell survival, in a dose-dependent manner. Such inhibitory effects included a significant decrease in the accumulation of lipid content by both a dramatic reduction of size and number of lipid droplets. This extract strongly attenuated the expression of PPARγ and HMGB2. It also inhibited the expression of CEBPα, FAS, and Akt without influencing Erk1/2 activities. Our findings suggest that specifically, the *Annona montana* ethyl acetate extract has a prominent inhibitory effect in cellular pathways of adipocyte differentiation by modulating specific gene expression, which is known to perform a pivotal role during adipogenesis.

## Introduction

The *Annonaceae* is a large family of plants containing approximately 130 genera and more than 2100 species. The fruits, seeds, leaves, and flowers of many of the *Annonaceae* plants are used in traditional medicines for the treatment of a variety of diseases. The Annona species are a potent source of a wide variety of secondary metabolites (e.g., acetogenins) belonging to several categories (Agostini et al., 1995; Coria-Téllez et al., 2018; Rauter et al., 2002; Moghadamtousi et al., 2015; Mootoo et al., 2000; Tundis et al., 2017; Wang et al., 2002; Wu et al, 1995).

*Annona (A.) montana,* the mountain soursop, is an edible, fibrous fruit in the *Annonaceae* family and is native to Central America, the Amazon, and Caribbean Islands. *A. montana* is also popularly known as guanabana or false graviola due to its similarity with graviola (*A. muricata*); however, *A. montana* exhibits glossy leaves and a more conspicuous spreading crown (Chatrou et al., 2012). In addition to *A. montana,* other species of the genus (e.g., *A. muricata, A. crassiflora, A. diversifolia,* and *A. squamosa*) are used in some countries as folk medicine based on their biological properties (e.g., anticancer, antiparasitic, antispasmodic, antidiarrheal, antiulcer, sedative, hypotensive, anthelmintic and antioxidant) (Adewole and Ojewole, 2008; Bailon-Moscosoa et al., 2016; Carballo et al., 2010; Degli Esposti et al., 1994; Dragano et al., 2010; Ponrasu and Suguna, 2012). Though, their efficacy as a medicine to treat disease, especially chronic illnesses, has yet to be validated scientifically.

One of the chronic, degenerative diseases is obesity, which has become one of the most prevalent metabolic diseases throughout the world. Obesity and its association with other illnesses and conditions have thus become significant burdens to global health pursuits in combating chronic disease and disability. Obesity, which stems from the excess storage of adipose tissue, is primarily caused by a significant increase in the amount of adipocytes due to the proliferation and differentiation of preadipocytes into mature adipocytes (Collaborators et al., 2017).

Several animal models, as well as *in vitro* systems, have been used extensively in diabetes research. Both mouse (3T3-L1) and human preadipocytes have been useful *in vitro* models for studying obesity, due to the observable accumulation of triglycerides upon differentiating in culture (Rosen and Spiegelman, 2000). Adipocyte differentiation is induced by either or both the expression and phosphorylation or either of specific genes, such as PPARγ, C-EBPα, AMPK, HMGB2, and Akt1 (Bost et al., 2005; Kemp et al., 2003; Lee et al., 2018; Rosen and Spiegelman, 2000; Spiegelman et al., 1993). Thus, several crude aqueous and organic solvents of plant extracts, as well as other pure active compounds that specifically target and inhibit adipogenesis, have been considered potentially promising treatments for obesity (Roberts et al., 2010). While many plants have been utilized to reduce the risk factors of developing these chronic, degenerative illnesses (Abrams et al., 2011; de Souza Mda et al., 2012; Parraga et al., 2011; Taleghani et al., 2020), there are no studies in the literature that associate the effect of extracts from *A. montana* aerial parts with the differentiation of preadipocyte to adipocyte.

Consequently, the purpose of this study was to examine the effects of *A. montana* leaves extracts on lipid droplet formation as well as the expression of several adipogenic markers for both human and mouse preadipocyte differentiation.

## Materials and Methods

### Reagents

Mouse embryo fibroblast 3T3-L1 (ATCC^®^CL173) and normal human primary subcutaneous preadipocytes (ATCC^®^ PCS210010) cells were purchased from American Type Culture Collection (ATCC, Manassas, VA). Dulbecco’s modified Eagle’s medium high glucose (DMEM), penicillin/streptomycin and L-glutamine were purchased from Mediatech, Inc. (Manassas, VA). PPARγ (Cat# 2443S), C/EBPα (Cat# 2295S), FAS (Cat# 4233S), HMGB2 (Cat# 14163S), AMPKα (Cat# 5832S) and phospho(p)-AMPKα (Cat# 50081S-Thr172), Akt1(Cat# 75692S) and p-Akt1 (Cat# 9018S-Ser473) antibodies were from Cell Signaling Technology (Boston, MA). GAPDH (Cat# G9545) antibodies were from Sigma-Aldrich (St. Louis, MO). All secondary antibodies (Cat# 305-035-045) were purchased from Jackson ImmunoResearch Laboratories (West Grove, PA). All other chemicals were obtained from Sigma-Aldrich unless otherwise stated.

### Preparation of the methanol extract

*Annona montana* leaves were collected from the Redland region in Miami, Florida, USA in 2019. The taxonomic identity was verified by the botanist at the herbarium of the Department of Biological Sciences at Florida International University (AMICTB1314). Leaves (2.5 g for both species, fresh weight) from one individual plant were cut into small pieces and extracted with methanol [i.e., *A. montana* methanol extract] (100 mL) in a 250 mL round bottom flask connected to a reflux condenser heated at 65°C for 45 min. The leaves were filtered, and plant material was washed twice with methanol (25 mL). The extract and washes were pooled. Evaporation of the methanol extract by rotavapor/desiccation yielded a residue of 540 mg for *A. montana*.

### Fractionation of the methanol extract

The individual plant methanol extract was partitioned among hexane, ethyl acetate and water. Briefly, leaves were extracted with methanol as indicated above. An aliquot (300 mg) of the residue obtained by methanol extraction was partitioned between methanol and hexane. The hexane was removed from the methanolic phase, then evaporated and concentrated by rotavapor/desiccation as previously described to obtain *A. montana* extract-hexane; the residue was partitioned between water (*A. montana* extract-water, and *A. montana* extract-ethyl acetate, respectively). These fractions were then individually evaporated and concentrated by rotavapor/desiccation as previously described (Galvis et al., 2011). The hexane, ethyl acetate, and water extracts yielded 24, 78, and 150 mg of residue for *A. montana,* representing approximately 84% of the initial methanol extract. All extracts were dissolved in dimethyl sulfoxide (DMSO) at a final concentration of 100 mg/ml plant extract.

### GC and GC/MS analyses

GC/MS determinations were carried out in a Hewlett Packard model 6890 instrument coupled to a Q-Mass 910 quadrupole selective detector (Perkin-Elmer) at 70 eV. A fused capillary column was used (DB-5MS, 30 m × 0.25 mm i.d.; film thickness 0.25 μm; J&W Scientific); injection port temperature, 230°C; splitless for 1 min then split ratio 1/10; detector temperature, 270°C; carrier gas, helium at 0.7 mL/minute; temperature program: 50–230°C linear increase at 3 °C/minute. Scanning speed was 2.48 scan/second with mass spectra recorded from 50 to 650 m/z. GC/FID determinations were performed on a Trace GC Ultra apparatus (Thermo Electron Corporation) equipped with an FID. The output was recorded using a ChromQuest version 4.1 data system. Analyses were performed on capillary columns DB-5MS at the conditions stated above (Adams, 2007; Araujo et al., 2007; Babushok et al., 2011).

### Cell culture and differentiation

Mouse 3T3-L1 cells were routinely maintained in Dulbecco’s modified Eagle’s medium (DMEM) high glucose supplemented with 10% fetal bovine serum (Invitrogen, Carlsbad, CA), 1% penicillin/streptomycin and 1% L-glutamine (growth media) in a humidified atmosphere of 5% CO_2_ at 37°C as described in the ATCC toolkit’s instructions (ATCC^®^ PCS210010™). Cells were split every 72 h and they were used up to 10 consecutively passages. To trigger differentiation cells were exposed to induction medium (IM) (growth media supplemented with 10 mg/mL insulin, 1 mM dexamethasone and 0.5 mM 3-isobutyl-1-methylxanthine [IBMX]) for the first two days, then fed with post-differentiation media (DMEM high glucose supplemented with 10 mg/mL insulin, 5% fetal bovine serum, 1% penicillin/streptomycin and 1% L-glutamine) for the next seven days. Cells were exposed to *A. montana* extracts throughout the entire differentiation process, unless otherwise indicated. Human primary subcutaneous preadipocytes were routinely maintained in fibroblast basal medium supplemented with the fibroblast growth kit-low (ATCC, Manassas, VA) containing 2% Fetal Bovine Serum, 5 ng/mL rh-FGF basic, 7.5 mM L-glutamine, 50 mg/mL ascorbic acid, 1 mg/mL hydrocortisone hemisuccinate, and 5 mg/mL rh-insulin as described in the ATCC’s instructions. Differentiation of human primary subcutaneous preadipocytes was induced by replacing basal media with initiation media for the first three days followed by maintenance media for eleven days; both the initiation and maintenance media were prepared from the adipocyte differentiation toolkit purchased from ATCC (ATCC^®^PCS500050, Manassas, VA). The human preadipocytes underwent *A. montana* extracts treatment throughout the entire differentiation process. The half maximal inhibitory concentration (IC_50_) is a quantitative measure that indicates how much of a particular inhibitory substance (e.g., plant extracts) is needed to inhibit, *in vitro,* a given biological process by 50% and it was essentially determined as described by Beck et al. (2012).

### Oil Red O staining, microscopy and image analysis

On the ninth day of experimentation, the cells were fixed with 10% formalin (Thermo Fisher Scientific, Inc., Pittsburgh, PA) in phosphate-buffered saline (PBS) at 4°C for 1 h. Oil Red O (Allied Chemical, Morristown, NJ) stock solution (0.6 g in 100 ml of 100% isopropanol) was diluted with 0.6 parts of double-distilled water, filtered, and added to the fixed cells for 15 min at room temperature. Cells were then washed with double-distilled water and analyzed under a Leica DM IRB inverted microscope. Photos of lipid droplets were taken with the digital Leica DC 500 camera. To quantify the lipid content from the lipids droplets stained with diazo dye Oil Red O (ORO). Then, ORO was eluted with 100% isopropanol for 10 min at 37°C, collected, and the optical density was measured at 540 nm. For image analysis, control and treated cells were processed as described above and the stained lipid droplets were visualized by a Leica DM IRB inverted microscope, photographed and digitized with a digital Leica DC 500 software at 400 x magnification. The number and size distribution of endosomes was analyzed by the NIH Image software, which can be accessed at the website (http://rsb.info.nih.gov/nihimage/). The total number of lipid droplets was quantified from, at least, 107 cells. The perimeters of 1484 lipid droplets from fortyfive ontrol cells and 992 lipid droplets from sixty two 40 mg/mL AMEaE-treated cells were obtained by using NIH Image software. The mean perimeter and the relative variance for the lipid droplets from control cells are 7.15 (2.27 diameter) and 0.41, while the corresponding values for lipids droplets the 40 mg/mL AMEaE-treated cells are 1.64 (0.52 diameter) and 0.35, respectively.

### Western blotting analysis

For the preparation of cell lysates, cell monolayers were washed with ice-cold lysis buffer (20 mM Tris-HCl pH 7.5, 150 mM NaCl, 1 mM Na_2_ EDTA, 1% NP40 and 0.1% Na Deoxycholate) containing both protease and phosphatase inhibitors. Lysates were collected by centrifugation, and protein concentrations were estimated by using the BCA protein assay (Thermo Fisher Scientific, Inc., Pittsburgh, PA) following the manufacturer’s instructions. Proteins were resolved by 10% SDS-PAGE and transferred to nitrocellulose membranes using a traditional wet transfer (100 V for 60 min), which were blocked in 5% non-fat dried milk in Tris-buffered saline (20 mM Tris, NaCl 150 mM, pH:8) with 0.1% Tween^®^ 20 detergent (TBS-T) buffer for 1 hr at room temperature, and then were incubated with primary antibodies against AMPKα (1:1000), p-AMPKα (1:1000), FAS (1:5000), Akt1 (1:2000), p-Akt1 (1:1000), HMGB2 (1:1000), PPARγ (1:1000), C-EBPα (1:2000), and GAPDH (1:5000, ab179467, Abcam) overnight at 4°C in 5% Bovine Serum Albumin (BSA)-TBS-T, followed by incubation with horseradish peroxidase (HRP)-conjugated secondary antibody (1:10,000) for 1 hr at room temperature in BSA-TBS-T. The protein bands were visualized using ECL detection reagent (Millipore, Billerica, MA, USA). In these cases, we carried out western blotting analysis with anti-phospho(p)AMPKα (or p-AKT1) antibody as indicated above. Then, the blot was stripped and reprobed with anti-total(t)AMPKα (or t-AKT1) antibody to ensure equal amount of protein in each lane. This stripped and reprobed processing was carried out by Thermo Scientific Restore Plus Western Blot Stripping Buffer kit (Cat# 46430) as indicated in the manual’s instructions. Relative levels of AMPKα and Akt1 proteins were determined by densitometry using the ratio of p-AMPKα to total(t)-AMPKα and p-Akt1 to t-Akt1, respectively. In this cases, Relative levels of FAS, HMGB2, PPARγ, and C-EBPα were determined using the ratio of FAS to GAPDH, HMGB2 to GAPDH, PPARγ to GAPDH and C-EBPα to GAPDH, respectively.

### Cell viability assay

The cell viability of 3T3-L1 cells was also analyzed using a trypan blue assay as reported in a previous study (Abood et al., 2018) with minor modifications. 3T3-L1 cells (1 × 10^4^ cells/mL) were incubated with *A. montana* extracts (0–40 mg/mL) for 9 day. The trypsinized cell suspension were immediately stained with 0.4% trypan blue for 2 min. The viable cells were counted by automated cell counter (BioRad, Hercules, CA, USA). The viability was expressed as the percentage ratio of the number of unstained cells relative to the total cells counted.

## Statistical Analysis

All experiments were done in triplicates and they were repeated at least three times. Values are represented as the standard error of the mean (S.E.M.) of triplicates and the statistical significance was analyzed by one-way ANOVA followed by Tukey’s *post hoc* test for multiple group comparisons. Two-tailed unpaired Student’s to test was performed if only two conditions were compared. Results with *P < 0.05 and **P < 0.01 were considered as statistically significant.

## Results

### Annona montana leaves’ extract inhibits differentiation of preadipocytes

**FIGURE 1.**
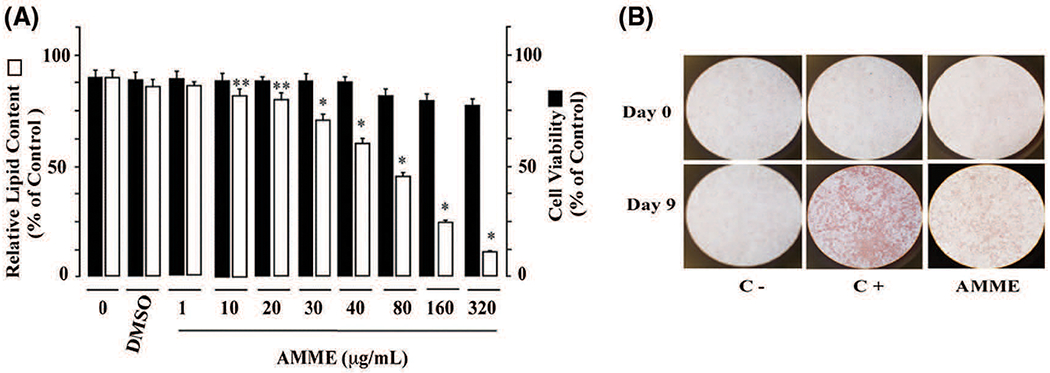
*Annona montana* methanol extract inhibited adipogenesis of 3T3-L1 preadipocytes without reducing cell viability. (A) 3T3-L1 preadipocytes were differentiated into adipocytes with induction media in the absence or the presence of various amounts of *A. montana* methanol extract (0 to 320 μg/ mL) as described in Material and Methods. Results were represented as relative lipid contents for adipocyte formation (open bars) and as percentage of control for cell viability (solid bars). Data represent the mean ± S.E.M. of three independent experiments. *P < 0.05 and **P < 0.01 by one-way ANOVA test compared to DMSO-control and only induction media-treated cells (0 μg/mL). (B) 3T3-L1 preadipocytes were monitored under a microscope and photographed after nine days (Day 9) from the onset of differentiation (Day 0). Vehicle only (C−), cells treated with induction media in the presence of DMSO-control (C+), or in the presence of 40 μg/mL *A. montana* methanol extract (AMME).

To examine the potential effect of *A. montana* leaves’ extract on the preadipocyte differentiation, we incubated 3T3-L1 with induction media in the presence of various concentrations of *A. montana* leaves methanol’s extract. In Fig. 1A, we show that the addition of *A. montana* methanol extract inhibited the lipid content in a dose-dependent manner with an IC_50_ of 84.9 ± 7.2 μg/mL. Furthermore, our experimental conditions demonstrate that the addition of DMSO (<0.2%) exclusively does not affect 3T3-L1 preadipocyte differentiation (Fig. 1A).

We were further interested in examining the effect of *A. montana* methanol extract on the viability of 3T3-L1 preadipocytes. Cells were incubated with induction media in the presence of 40 μg/mL *A. montana* methanol extract throughout the entire differentiation period. Cell viability was measured using the trypan blue exclusion assay, as described in Material and Methods. In Fig. 1A, we show that cell viability was not affected as compared with control cells (Compare viability of control cells (98 ± 2%) with the viability of cells treated with 40 μg/mL *A. montana* methanol (95 ± 5%). However, at increasing concentrations of *A. montana* methanol extract beyond 40 μg/mL, 3T3-L1 preadipocytes detached from the plate, which was coupled with a reduction in the trypan blue exclusion assay (Fig. 1A). These results suggest that, up to a maximum concentration of 40 μg/mL, *A. montana* methanol extract has a strong inhibitory activity of 3T3-L1 preadipocyte differentiation without significant effect on cell viability.

Adipocyte differentiation was also monitored by the formation of intracellular lipid droplets (Rosen and Spiegelman, 2000). As aforementioned, 3T3-L1 preadipocytes were cultivated and induced to differentiate into adipocytes with induction media in the absence or presence of 40 μg/mL *A. montana* methanol extract. At the end of the differentiation process, Oil Red O staining showed an abundant number of lipid droplets, which suggested a significant lipid accumulation in untreated differentiated cells. Moreover, lipid droplets were not observed in untreated non-differentiated cells. However, the formation of lipid droplets was inhibited by 40 μg/mL *A. montana* methanol extract treatment (Fig. 1B). The quantitative measurement of lipid content further supported these observations by determining the absorbance at 540 nm.

**FIGURE 2.**
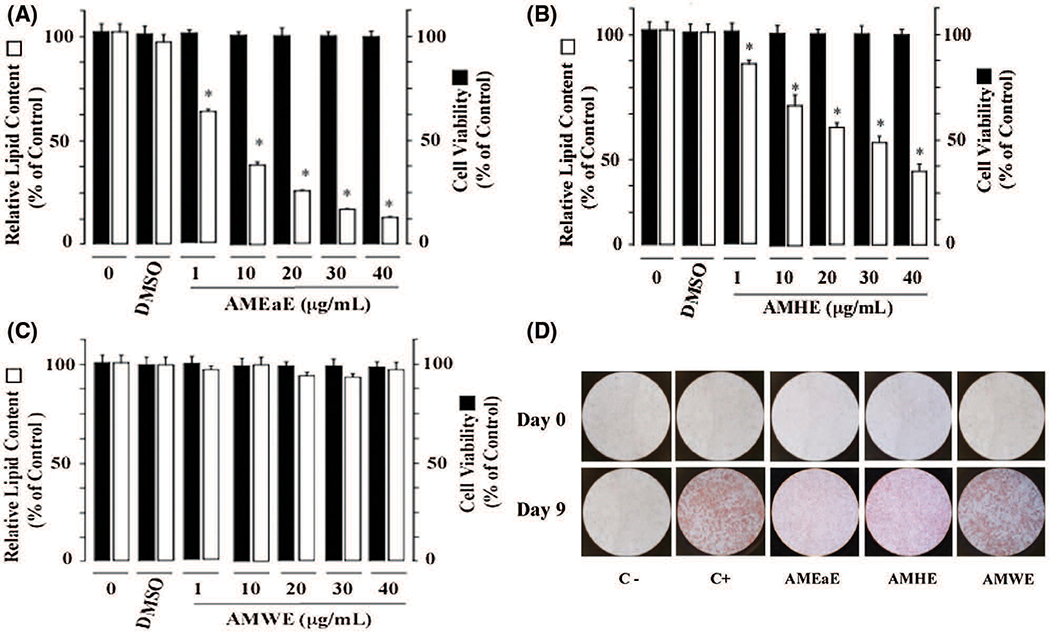
*Annona montana* extracts have selective inhibitory effects on the adipogenesis of 3T3-L1 preadipocytes without reducing cell viability. 3T3-L1 preadipocytes were differentiated into adipocytes with induction media in the absence or the presence of DMSO-control, or of various amounts (0 to 40 μg/ mL) of *A. montana* extracts (e.g., (A) *A. montana* ethyl acetate extract (AMEaE), (B) *A. montana* hexane extract (AMHE), and (C) *A. montana* water extract (AMWE) as described in Material and Methods. Results were represented as relative lipid contents. Data represent the mean ± S.E.M. of three independent experiments. *P < 0.05 and **P < 0.01 by one-way ANOVA test compared to DMSO-control and only induction media-treated cells. (D) 3T3-L1 preadipocytes were monitored under a microscope and photographed after nine days (Day 9) from the onset of differentiation (Day 0). Vehicle only (C−), cells treated with induction media in the presence of DMSO-control (C+), or in the presence of 40 μg/ mL *A. montana* extracts as indicated in the figure.

Our data indicate that the methanol extract of *A. montana* exhibited inhibition of adipocyte differentiation with an IC_50_ of 84.9 ± 7.2 μg/mL. Thus, to gain information about the nature of the active compounds in *A. montana* leaves, the methanol extract, a complex mixture of compounds, was further fractionated as described in the Materials and Methods. Three fractions (named as hexane, ethyl acetate, and water fractions) were obtained. The *A. montana* ethyl acetate fraction exhibited the most potent inhibitory effect (IC_50_ 7.2 ± 3.1 μg/mL) on cell differentiation as compared with the *A. montana* hexane fraction, which showed an inhibition of the preadipocyte differentiation with IC_50_ 50.9 ± 4.3 μg/mL (Figs. 2A and 2B). Interestingly, the aqueous fraction did not show any effect at all (Fig. 2C). As expected, these fractions did not show a significant reduction in the trypan blue exclusion assay (i.e., viability of cells treated by *A. montana* ethyl acetate fraction 82 ± 5%, by *A. montana* hexane fraction 93 ± 4%, or by *A. montana* aqueous fraction 97 ± 4% as compared with 92 ± 8% control cells). These observations on the *A. montana* extracts’ inhibitory effects on differentiation were further supported with the quantitative measurement of lipid content by determining the absorbance at 540 nm when cells were treated with different *A. montana* extracts. Additionally, *A. montana* ethyl acetate and hexane extracts treatment significantly inhibited adipogenic morphology, but not in the presence of *A. montana* aqueous extract (i.e., transition from a fibroblast-like shape to an increasingly round appearance with an accumulation of lipid droplets (Fig. 2D).

**FIGURE 3.**
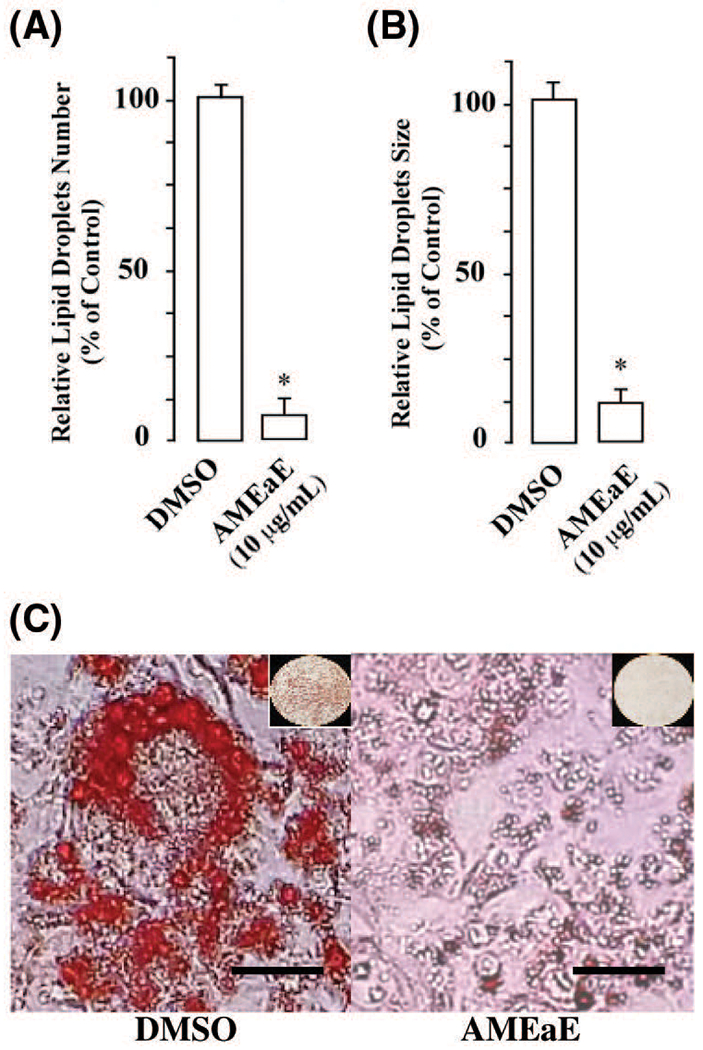
*Annona montana* ethyl acetate extract decreased the number and the size of lipid droplets during adipogenesis. Preadipocytes were incubated with induction medium in the presence of DMSO-control or containing 10 μg/mL *A. montana* ethyl acetate extract (AMEaE) for nine days. After differentiation, the cells were stained with Oil Red O and then photographed. (A) The total number of lipid droplets was quantified from at least 100 cells. *P < 0.05 DMSO-control *vs*. AMEaE-treatment. (B) The perimeters of 1584 lipid droplets from forty-eight control cells and 1092 lipid droplets from fifty-two of AMEaE-treated cells were obtained with the use of NIH Image software. Lipid droplets have a diameter in the range of 0.81 to 3.90 μm. *P < 0.05 DMSO-control *vs.* AMEaE-treatment. Results were represented as percentage of DMSO-control. (C) 3T3-L1 preadipocytes were monitored under a microscope and photographed after nine days from the onset of differentiation. Cells treated with induction media in the presence of DMSO-control, or in the presence of 10 μg/mL AMEaE. Scale bars: 10 μm. Insets show the field of vision.

Consistent with these observations, we found that the *A. montana* ethyl acetate treatment decreased both the number and size of lipid droplets. Specifically, we observed more than 93% reduction in the number of lipid droplets and more than 87% reduction in the size of lipid droplets (Figs. 3A and 3B). Analysis of the distribution of the numbers of lipid droplet sizes showed a distinct shift of the lipid droplet size toward smaller lipid droplets following incubation with *A. montana* ethyl acetate extract. The mean perimeter and the relative variance for the lipid droplets from control cells are 8.54 (3.90 diameter) and 0.41, while the corresponding values for lipids droplets of 10 μg/mL *A. montana* ethyl acetate extract-treated cells are 1.74 (0.81 diameter) and 0.35, respectively (Fig. 3C). Furthermore, human primary subcutaneous preadipocytes were also subjected to *A. montana* ethyl acetate treatment at several concentrations (i.e., 0.25 to 40 μg/mL). Observations under the microscope and Oil Red O analysis indicated a significant inhibition (more than 90% inhibition) of differentiation of the preadipocytes into adipocytes with increasing concentration of *A. montana* ethyl extract without affecting cell viability (Supplemental Fig. 1A). Compared to the positive controls, *A. montana* ethyl acetate extract (i.e., 1 μg/mL) also decreased lipid number and size in human preadipocytes (Supplemental Fig. 1B). These results suggest that, up to a maximum concentration of 10 μg/mL, *A. montana* ethyl acetate extract has a strong inhibitory activity of human preadipocyte differentiation without significant effect on cell viability.

**FIGURE 4.**
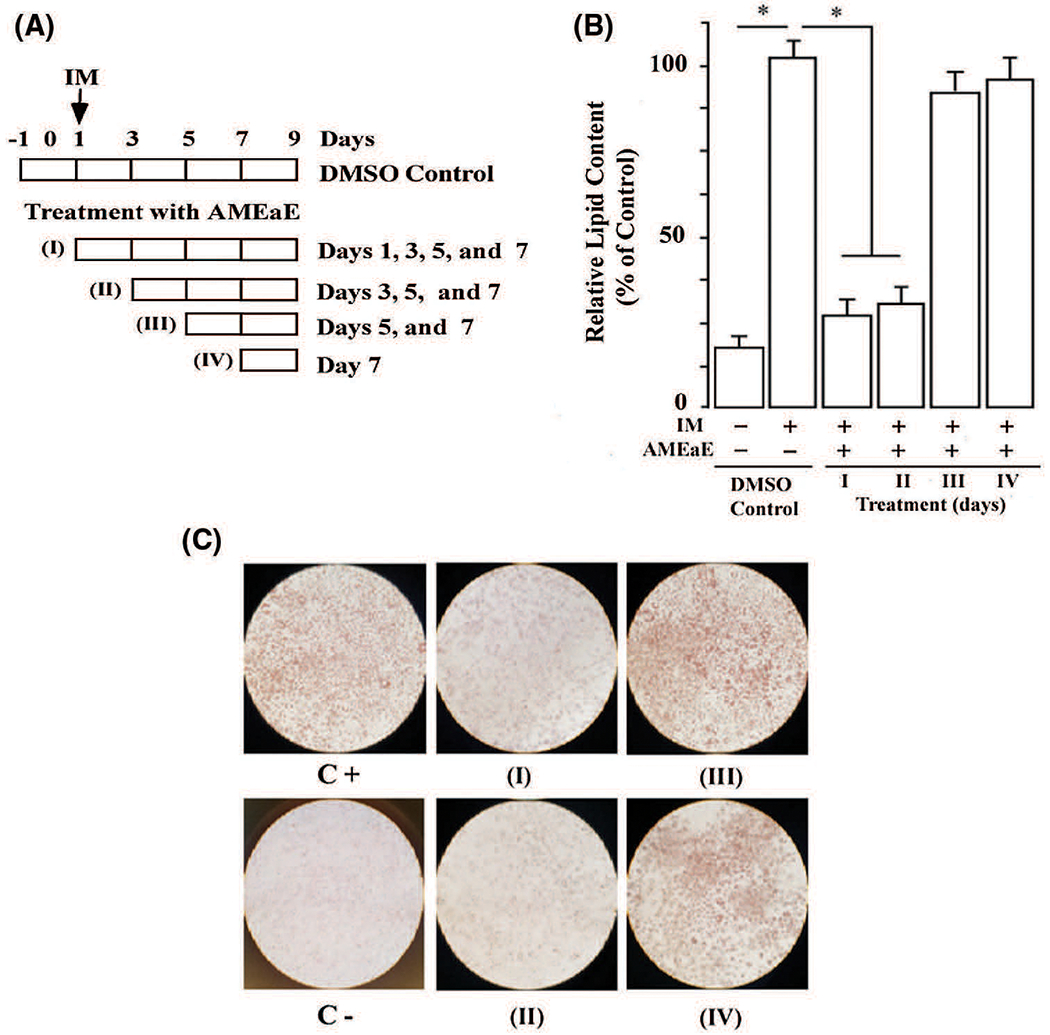
*Annona montana* ethyl acetate extract blocked adipocyte differentiation in a time-dependent manner. (A) 3T3-L1 preadipocyte cells incubated with induction medium alone (IM) in the presence of DMSO-control or treated with 10 μg/mL *A. montana* ethyl acetate extract (AMEaE) for the time indicated in the schematic representation of the experiment. (B) In each treatment (i.e., I, II, III and IV), the accumulation of lipid droplets was measured by the incorporation of Oil Red O as described in Material and Methods. Data represent the mean ± S.E.M. of three independent experiments. *P < 0.05 by two Student’s *t*-test compared to DMSO-control induction media-treated cells (control, C+). Vehicle only (C−) was also shown. (C) 3T3-L1 preadipocytes were monitored under a microscope and photographed after nine days (Day 9) from the onset of differentiation (Day 0). Cells treated in the absence (control, C−) or in the presence of induction media with DMSO (Control, C+), or with 10 μg/mL AMEaE for each specific treatment (I to IV) as indicated in the figure.

To identify the critical stage of adipogenic differentiation affected by *A. montana* ethyl acetate extract treatment, differentiating 3T3-L1 preadipocytes were treated with 10 μg/mL *A. montana* ethyl acetate extract at various time points during adipogenic differentiation, as illustrated in Fig. 4A. Thus, we also took into consideration the possibility that the extent of inhibition is dependent on the timing of *A. montana* ethyl acetate extract addition. Consequently, *A. montana* ethyl acetate extract was added in discrete periods during differentiation (Fig. 4A). We observed a significant reduction in the differentiation of 3T3-L1 preadipocytes when compared with DMSO-control cells upon early *A. montana* ethyl acetate extract addition, corresponding to days 1 or 3 of treatment (e.g., treatments I and II, Fig. 4B). However, this inhibitory effect was not apparent when *A. montana* ethyl acetate was added on day 5 or day 7 of postinduction (e.g., treatments III and IV, Fig. 4B). Observations under the microscope also confirmed significant inhibition of the lipids droplets during treatment for days 1 and 3, but not for days 5 and 7, respectively (Fig. 4C). These results further demonstrate that *A. montana* ethyl acetate may show a selective and early adipocyte gene expression during *in vitro* differentiation. Taken together, these results suggest that *A. montana* ethyl acetate extract could inhibit differentiation of mouse 3T3-L1 and human preadipocytes by affecting the expression of one or more transcriptional factors (i.e., inhibition expression of PPARγ), which would be consistent with the robust effect of *A. montana* ethyl acetate extract on the number and size of the lipid droplets.

### Annona montana *ethyl acetate extract inhibits key adipogenic transcriptional factors*

A coordinated network of expression and activation of key transcriptional factors and signaling molecules is critical for adipogenesis (Rosen and Spiegelman, 2000). PPARγ and C-EBPα are two major adipogenic transcription factors, which are required during the early steps of preadipocytes differentiation (Rosen et al., 2002). To investigate whether *A. montana* ethyl acetate extract affects the expression of PPARγ and C-EBPα, we incubated 3T3-L1 preadipocytes with induction media in the absence or presence of 10 μg/mL *A. montana* ethyl acetate extract. We then harvested the cells at day 9 for western blotting analysis using anti-PPARγ and anti-C/EBPα antibodies, respectively.

**FIGURE 5.**
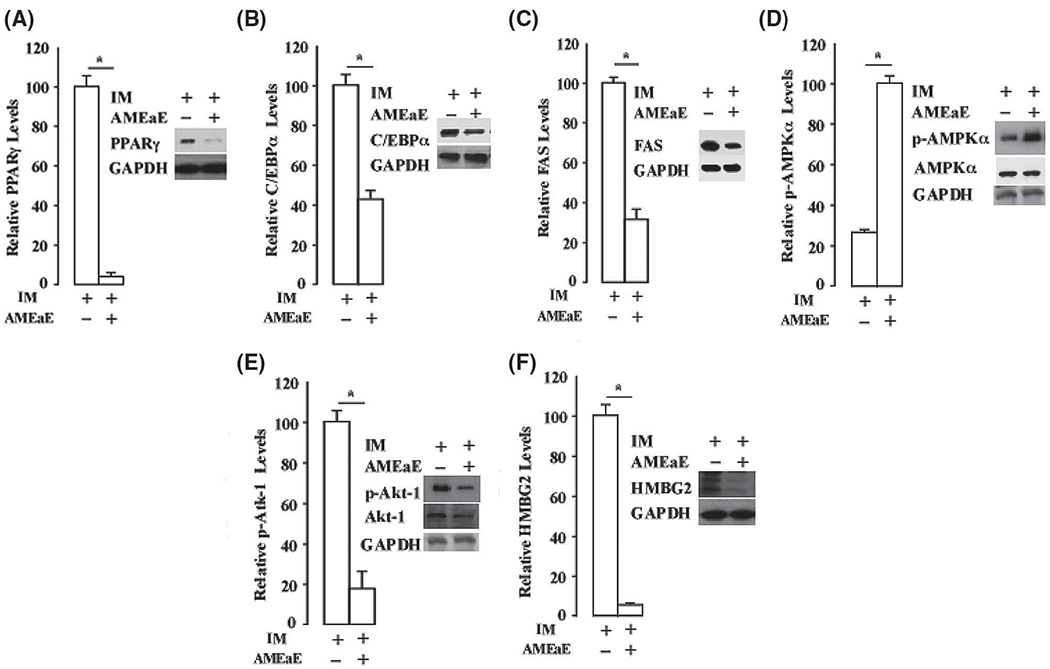
*Annona montana* ethyl acetate extract strongly attenuated the expression of PPARγ during 3T3-L1 preadipocyte differentiation. 3T3-L1 preadipocyte differentiation was induced by induction media (IM) alone in the presence of DMSO-control, or in the absence or the presence of 10 μg/mL *A. montana* ethyl acetate extract (AMEaE) (inset: −AMEaE, +AMEaE). Total protein extracts were prepared on Day 9 from each sample, and probed with antibodies specific to (A–B) PPARγ, C-EBPα and GAPDH, (C) FAS and GAPDH, (D) p-AMPKα, t-AMPKα, (E) p-Akt1, t-Akt1 and (F) HMGB2, respectively. GAPDH was used as loading control. Relative levels of proteins were determined by densitometry as described in Material and Methods. Data represent the mean ± S.E.M. of three independent experiments. *P < 0.05 by two student’s *t*-tests compared to DMSO IM-treated cells.

In Figs. 5A and 5B, we showed that the addition of *A. montana* ethyl acetate extract attenuated the expression of PPARγ and C/EBPα as well as other adipogenic factors (e.g., FAS, Fig. 5C). We also examined whether the addition of *A. montana* ethyl acetate extract affected the phosphorylation of AMPKα during 3T3-L1 preadipocyte differentiation. This result shows that phosphorylation of AMPKα (p-AMPKα) was not inhibited by the addition of *A. montana* ethyl acetate extract.

In contrast, *A. montana* ethyl acetate extract enhanced phosphorylation of AMPKα (Fig. 5D). In addition, we also found that the level of total expression of AMPKα was not affected in the presence or absence of *A. montana* ethyl acetate extract. Therefore, these results indicated that the level of phosphorylated AMPKα increased in the presence of *A. montana* ethyl acetate extract. However, the expression of PPARγ was inhibited by more than 95% upon the addition of *A. montana* ethyl acetate extract.

We also examined the effect of *A. montana* ethyl acetate extract on the phosphorylation of Akt1 and Erk1/2 proteins. Surprisingly, we found that the addition of *A. montana* ethyl acetate extract attenuated phosphorylation of Akt1 (Fig. 5E), without inhibiting the phosphorylation of Erk1/2 (data not shown). Interestingly, the total level of expression of AkT-1 and AMPKα were not affected by the addition of *A. montana* ethyl acetate extract (Figs. 5D and 5E), We also tested whether the addition of *A. montana* ethyl acetate extract affected the expression of HMGB2 factor, an early stage adipogenic factor (Lee et al., 2018), during 3T3-L1 preadipocyte differentiation. The result indicated that the addition of this extract also inhibited the expression of HMGB2 (Fig. 5F). Altogether, these data suggest that the *A. montana* ethyl acetate extract strongly and selectively blocks the expression of PPARγ and HMGB2, and also decreases the expression of FAS and Akt1 during the differentiation of 3T3-L1 preadipocytes, which indicates that *A. montana* ethyl acetate extract may affect early adipocyte gene expression during *in vitro* differentiation.

### Chemical Composition of the Annona montana ethyl acetate extract

Chemical studies with species of the Annona genus have reported on the essential oils found in the fruits, whose composition is predominantly of monoterpenes and sesquiterpenes and alkaloids. Because many *Annonaceae* fruits are edible, they are the predominant focus of many studies. On the contrary, few studies have been performed on the leaves. Thus, we conducted a Gas Chromatography-Mass Spectrometry (GC-MS) analysis as described in Material and Methods to gain more information about the composition of the constituents, specifically in the *A. montana* ethyl acetate extract. Our results on *A. montana* ethyl acetate leaves extract indicated that twenty-two compounds out of a total of twenty-seven compounds were identified, (Table 1 and Supplemental Table 1) in a complex mixture of sesquiterpenes (i.e., α-copaene, β-elemene, β-caryophyllene and δ-cadinene), fatty acids (i.e., Linoleic acid, Octadecanoic acid), phytosterols (i.e., Campesterol, γ-Sitosterol and Stigmasta-5,22-dien-3-ol), hydrocarbons (i.e., 1-Hexacosanol, 9-Hexacosene and 1-Docosanethiol), β-tocopherol, 1,22-Docosanediol, and Z-11(13-Methyl) tetradecen-1-ol acetate. Interestingly, some of these compounds have been reported to affect some cellular mechanisms of cell proliferation and apoptosis in cancer cells as well as they may reduce hyperglycemia in Streptozotocin-induced diabetic rats (Balamurugan et al., 2011; Sundarraj et al., 2012).

## Discussion

Several studies have determined that the utilization of both leaves and fruits of medicinal plants can lessen the occurrence of risk factors involved in obesity and cancer (Agostini et al., 1995; Coria-Téllez et al., 2018; Matsuda et al., 2014; Meneguetti et al., 2011). Our results demonstrate that *A. montana’s* leaves extracts studied in this research may be beneficial for the control and prevention of fat formation and its risk factors, since the extracts reduced lipid droplet formation in adipocytes by affecting several adipogenic factors.

Two major adipogenic transcription factors, PPARγ. and C-EBPα, work together to induce the expression of adipocyte-specific genes, which are involved in the switch between proliferation and differentiation in cells (Rosen et al, 2002). Studies on adipogenic cell lines have also shown that HMGB2 is expressed during adipogenesis and seems to be upstream of PPARγ and C-EBPα (Lee et al., 2018). The activity of the critical enzyme fatty acid synthetase facilitates the formation of cytoplasmic triglyceride, the end-product of the lipogenic pathway during the differentiation of 3T3-L1 cells (Student et al., 1980). Furthermore, both PI3K/Akt and Ras/Erk signaling pathways are critical and may affect adipogenesis in distinct ways depending on their appropriate time of activation upon insulin stimulation during the differentiation process (Kim and Chen, 2004; Prusty et al, 2002).

Our *in vitro* data showed that PPARγ and HMGB2 were among the genes that were strongly downregulated by the addition of *A. montana* ethyl acetate extract. The extract also inhibited the expression of C-EBPα and Akt but to a lesser extent. In contrast, Erk activity was not affected in the presence of *A. montana* ethyl acetate extract even at higher concentrations (data not show). Treatment of 3T3-L1 preadipocytes with *A. montana* ethyl acetate extract dramatically reduced protein expression of PPARγ, which is strictly concordant with the appearance of cytoplasmic lipid droplets. These data suggest that this extract may target specific pathways related to the expression of PPARγ. and, in lesser extension, the expression C-EBPα, due to the extract’s strong inhibition of PPARγ. Furthermore, our observations showed that the *A. montana* ethyl acetate extract might have selective effects on adipocyte differentiation since Akt activity, which are thought to be sensitive to insulin stimulation, were also affected by the addition of *A. montana* ethyl acetate extract. Consistent with this result, Akt has been demonstrated to regulate adipogenesis *via* phosphorylation by inactivating Foxo1, in which Foxo1 is known to regulate PPARγ activity directly (Nakae et al., 2003). Thus, it is reasonable to consider that *A. montana* ethyl acetate extract may selectively block pathways specific to these two signaling molecules (i.e., AKT and PPARγ) by regulating adipocytic differentiation containing the adipocyte-specific factor.

The effects of other *Annona’s species* on several biological assays have also been evaluated. Aqueous and methanol extracts from *A. muricata* leaves showed both hypoglycemic and antioxidant effects on diabetic and on Streptozotocin-induced diabetic rats (Adeyemi et al., 2008). Remarkably, even though *A. muricata, A. squamosa,* and *A. reticulata* presented antioxidant activities, it was concluded that *A. muricata* had more potent antioxidant activities in several *in vitro* studies (Baskar et al., 2007). Studies have also shown that the administration of extracts prepared from *A. squamosa* produced an inhibitory effect on glycemic and lipemic indices as well as on the levels of the antioxidant enzymes (i.e., glutathione reductase) (Gupta et al., 2005; Kaleem et al., 2006). However, studies on *A. crassiflora* have shown minimal effects on lipemia and glycemia in rats (Dragano et al., 2010). Ethanolic extracts from *A. cuneate, A. coriaceae,* and *A. senegalensis* showed a strong antiinflammatory effect, which is likely due to the high content in the variety of polyphenolic components and vitamins (i.e., Vitamin C). These effects may be attributed to the presence of compounds such as polyphenols, flavonoids, steroids, and a series of secondary metabolites that can contribute to the reduction of glycemia and antiinflammatory effects (Barbalho et al., 2011; Coria-Téllez et al., 2018; Dragano et al., 2010; Grover et al., 2011), which could increase the range of therapeutic effects of this species.

Our results indicated the presence of alkaloids, acetogenins, phenols, carotenoids, amides, cylopeptides, sesquiterpenes, and aromatic and aliphatic esters in the *A. montana* ethyl acetate extract. Some of these compounds have also been identified in *A. muricata* extracts (Coria-Téllez et al., 2018). Additionally, we have described the presence of several sesquiterpenes (i.e., α-Copaene, β-Caryophyllene, β-Elemane, and δ-Cadinene) in the ethyl acetate fraction. Interestingly, some of these terpenes have been implicated in the inhibition of proliferation of several types of cancer cells and in PARP expression (Hui et al., 2015; Matsuda et al., 2014), which can help us to explain, at least in part, the inhibitory effect of *A. montana* extracts.

## Conclusion

These results demonstrate that *A. montana* leaves extracts strongly inhibited *in vitro* differentiation of mouse and human preadipocytes, which may indicate useful applications to human health by helping to lower the incidence of chronic degenerative diseases. Future investigations on plant extracts’ effects on adipogenesis would also expand from the inclusion of other species of the genus (e.g., *A. muricata, A. crassiflora, A. diversifolia,* and *A. squamosa*).

## Supplementary Material

Supplemental Figure 1

Supplemental Table 1

## Figures and Tables

**Table 1:** Chemical composition and percentage of compounds from the leaves of *Annona montana* ethyl acetate extract

		Rt/min	Area
1	α-Copaene	15.03	0.9
2	β-Elemene	15.42	0.7
3	β-Caryophyllene	16.15	0.4
4	δ-Cadinene	17.65	2.1
5	Unknown	24.82	0.5
6	cis-pinane	25.46	4.7
7	Unknown	25.59	4.3
8	1,4-Eicosadiene	26.32	1.3
9	n-Hexadecanoic acid	27.91	6.6
10	Phytol	30.70	1.8
11	Linoleic acid	31.06	7.2
12	Octadecanoic acid	31.56	1.5
13	1-Docosanethiol	37.00	3.5
14	Octadecanal	37.60	1.0
15	Docosanoic Acid	38.12	0.9
16	1,22-Docosanediol	39.13	0.6
17	1-Hexacosene	42.86	3.6
18	Unknown	43.15	1.4
19	Z-11(13-Methyl)tetradecen-1-ol acetate	44.73	1.2
20	β-tocopherol	44.95	2.3
21	Unknown	45.12	19.0
22	1-Hexacosanol	45.53	7.4
23	Campesterol	47.20	3.1
24	Unknown	47.26	2.0
25	Stigmasta-5,22-dien-3-ol	47.6	2.8
26	9-Hexacosene	48	2.8
27	γ-Sitosterol	48.32	11
	**Total**		**94.6**

Note: RT: Retention time. The compounds were identified using the National Institute of Standards and Technology (NIST) EPA NIH mass spectral database program (NIST v17; version 2.3).
