# Supplementary figures and images for: Anti-lipid droplets accumulation effect of Annona montana (mountain soursop) leaves extract on differentiation of preadipocytes
Source: Biocell. Author manuscript; Available in PMC 2022 Jan 1. (PMC8713457; doi:10.32604/biocell.2022.014009)

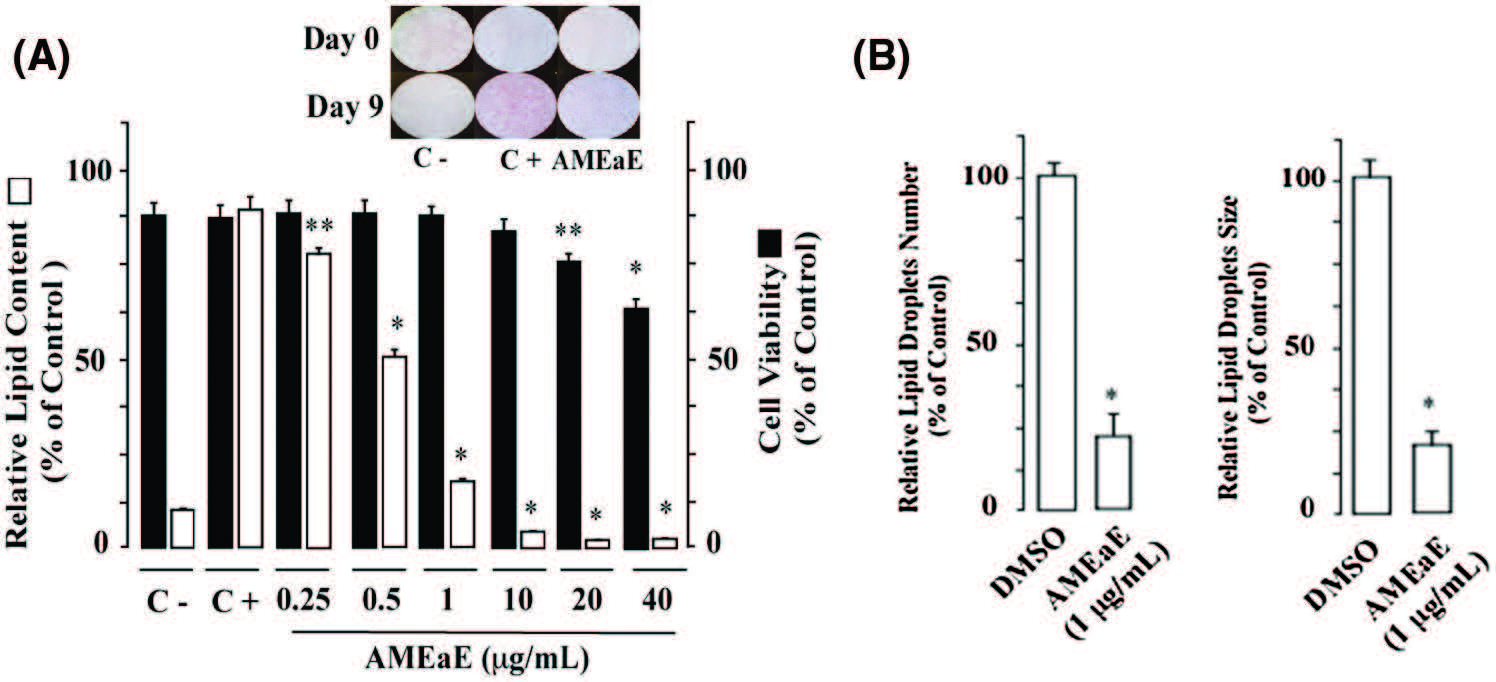

Supplement: Supplemental Figure 1 [file NIHMS1757872-supplement-Supplemental_Figure_1.jpg]
