# Supplementary material for: Anti-lipid droplets accumulation effect of Annona montana (mountain soursop) leaves extract on differentiation of preadipocytes
Source: Biocell. Author manuscript; Available in PMC 2022 Jan 1. (PMC8713457; doi:10.32604/biocell.2022.014009)
Supplement: Supplemental Table 1 [file NIHMS1757872-supplement-Supplemental_Table_1.docx]

**Supplemental Table 1. Chemical composition and percentage of compounds from the leaves of**

***Annona montana* ethyl acetate extraction**

**_________________________________________________________________________________**

**Component % Ri^a^ Ri^b^ Identification^c^**

**_________________________________________________________________________________**

α-Copaene 1.1 1375 1368 1,2,3

δ-Elemene 0.9 1338 1327 1,2,3

β-Caryophyllene 0.4 1588 1598 1,2,3

δ-Cadinene 2.1 1755 1722 1,2,3

cis-Pinane 5.1 1002 1018 1,2

1,4-Eicosadiene 1.1 2007 1997 1,2

n-Hexadecanoic acid 7.6 2913 2862 1,2

Phytol 2.1 2613 2619 1,2

Linoleic acid 2.1 3148 3201 1,2,3

Octadecanoic acid 1.7 2158 2142 1,2

1-Docosanethiol 4.1 1518 1536 1,2

Octadecane 1.1 1357 1387 1,2

Docosanoic Acid 0.7 2560 2490 1,2

1,22-Docosanediol 0.4 1518 1536 1,2

1-Hexacosene 4.1 2593 2578 1,2

Z-11(13-Methyl)tetradecen-1-ol acetate 1.1 1822 1875 1,2

β-tocopherol 40.1 3075 3068 1,2,3

1-Hexacosanol 3.2 2932 2905 1,2

Campesterol 3.1 3305 3354 1,2,3

Stigmasta-5,22-dien-3-ol 3.3 2739 2810 1,2

γ-Sitosterol 12.1 3790 3798 1,2,3

**____________________________________________________________________________**

RI^a^: retention indices (DB-5 column) calibrated against n-alkanes; RI^b^: reference retention indices

recorded in the literature [Adams 2007; Babushok *et al*, 2011; Araujo *et al* 2007]; Identification^c^ 1 = Kovats retention index, 2 = mass spectrum, 3 = co-injection with authentic compound.
